# Reconsidering Sex‐Specific Alcohol Guidelines for Atrial Fibrillation Prevention: Caution Against Premature Policy Change

**DOI:** 10.1002/joa3.70184

**Published:** 2025-09-10

**Authors:** Brijesh Sathian, Javed Iqbal, Syed Muhammad Ali

**Affiliations:** ^1^ Hamad Medical Corporation Doha Qatar

**Keywords:** alcohol consumption, atrial fibrillation, sex differences

Dear Editor,

Matsunaga‐Lee et al. are to be congratulated on the use of a large inpatient cohort to investigate the association between alcohol consumption and atrial fibrillation risk in men and women. Their results, especially the lack of a protective low‐dose threshold in men, are thought‐provoking and call into question the sex‐specific drinking limits suggested by present guidelines. A number of methodological issues need to be addressed before conclusions can be firmly drawn for public health policy [1].

First, the cross‐sectional design limits causal inference by nature. Prevalent compared to incident AF, it introduces survival bias and reverse causation. AF patients might reduce alcohol consumption following diagnosis, causing misclassification of exposure, a well‐recognized limitation in lifestyle‐disease epidemiology [2].

Second, the sole use of an inpatient sample creates issues with selection bias. Populations admitted to hospital settings are systematically different from community samples regarding comorbidity profiles, health behavior, and socioeconomic status [3]. These differences may interact with both sex and alcohol use and skew observed relationships.

Third, the lack of beverage‐type information is a substantial limitation. Previous studies have established beverage‐specific relationships, with AF beer and spirits tending to show greater risk than wine even at similar ethanol doses [4, 5]. Given the sex‐related differences in beverage choice reported in Japanese and Western cohorts, failure to adjust for beverage type has the potential to confound the seeming sex interaction.

Fourth, even though the authors accounted for important clinical covariates, residual confounding is still possible. Important covariates like smoking, physical activity, socioeconomic status, and biomarkers (e.g., NT‐proBNP, CRP) were not available. Without them, it is not easy to rule out other explanations for the observed association between low‐level alcohol consumption and AF in men.

Lastly, the analysis seems to interpret the observed sex interaction as evidence of harmonious alcohol limits between the sexes at ≤ 20 g/day. Although this is an interesting hypothesis, it is too early on the basis of the study's design and the potential for differential misclassification by sex in self‐reported alcohol consumption [6]. Prospective, population‐based research with repeated, validated exposure measures is required to support these trends prior to updating national guidelines. Future studies should examine beverage types to determine whether some drinks are disproportionately linked to AF risk in both sexes, as well as cross‐national comparisons to capture cultural and genetic variations. Stronger evidence for improving sex‐specific guidelines would come from such methods.

In conclusion, Matsunaga‐Lee et al. offer useful data, but the design, sampling, and exposure characterization limitations moderate the quality of the causal inferences. The paper should be considered hypothesis‐generating and not practice‐changing, and future studies addressing these limitations will be important to inform sex‐specific AF prevention strategy development.

## Disclosure

*Declaration of AI Content*: This article was not generated by AI tools. While AI was utilized to enhance the professionalism and readability of the content, it was not used extensively to the extent that the work appears AI‐generated. The primary content, analysis, and conclusions are the result of the authors' original work.

## Ethics Statement

The authors have nothing to report.

## Conflicts of Interest

The authors declare no conflicts of interest.
